# Operando ZnO recrystallization for efficient quantum-dot light-emitting diodes

**DOI:** 10.1038/s41377-025-01867-1

**Published:** 2025-05-15

**Authors:** Song Wang, Shihao Liu, Ting Wang, Jialin Bai, Jingyu Peng, Hanzhuang Zhang, Wenfa Xie, Wenyu Ji

**Affiliations:** 1 Key Lab of Physics and Technology for Advanced Batteries (Ministry of Education), College of Physics, Jilin University, Changchun, 130012 China; 2 State Key Laboratory of Integrated Optoelectronics, College of Electronic Science and Engineering, Jilin University, Changchun, 130012 China; 3 Key Laboratory of Functional Materials Physics and Chemistry of the Ministry of Education, Jilin Normal University, Changchun, 130103 China

**Keywords:** Quantum dots, Electronics, photonics and device physics

## Abstract

ZnO nanoparticles (NPs) play a crucial role in advancing quantum-dot light-emitting diodes (QLEDs) because of their excellent electron transport properties. While the conductivity of ZnO is determined by both the density and mobility of charge carriers, a previously overlooked problem is that excessive carrier density in ZnO can lead to nonradiative Auger recombination at the quantum-dot/ZnO interface. An ideal electron transport layer should possess both high mobility and low carrier density. Here, we achieve such transport properties in ZnO NP films through operando recrystallization, a process triggered by the diffusion of Al ions from the cathode under acidic conditions. This diffusion induces the coalescence of neighboring ZnO NPs, forming defect-passivated, long-range ZnO crystals. When used as the electron transport layer in QLEDs, recrystallized ZnO NPs enhance the external quantum efficiency from 17.2% to 33.7% compared with devices with conventional ZnO electron transport layers. These findings offer valuable insights into the development of charge transport materials for high-performance optoelectronic devices.

## Abstract (web summary)

Operando recrystallization of ZnO NP films passivates defects and boosts electron mobility, enhancing radiative recombination and electron injection in QDs to achieve 33.7% EQE.

## Graphical abstract

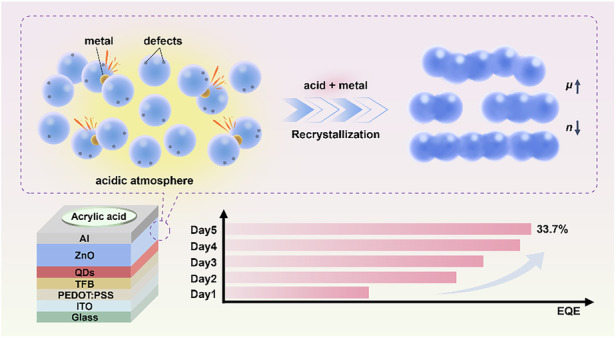

## Introduction

Quantum-dot light-emitting diodes (QLEDs) are promising candidates for next-generation displays and lighting technology^1–4^. Significant progress in QLED performance has been made since the introduction of ZnO nanoparticles (NPs) as the electron-transport layer (ETL)^5–8^. However, some fundamental physical issues in QLEDs remain unresolved. One such phenomenon is “positive ageing,” where the external quantum efficiency (EQE) of QLEDs increases abnormally with operational or storage time, which is attributed to operando chemical reactions of ZnO NPs^9,10^. The exceptional performance of hybrid QLEDs is often related to the electronic conduction properties of the ZnO ETL^11,12^, with conductivity (*σ*) being determined by the charge carrier density (*n*) and mobility (*μ*). However, a previously overlooked issue is that a high charge carrier density leads to a high electron density in the quantum dot (QD) emission layer, resulting in nonradiative Auger recombination that limits device efficiency. Thus, the impact of *n* and *μ*, rather than that of *σ*, on exciton recombination must be carefully considered to understand the performance of QLEDs.

The high density of charge carriers in ZnO NP films, induced by surface defects, is primarily responsible for good electronic conduction, but this also leads to significant nonradiative recombination and poor device efficiency^13–15^. To achieve optimal performance, positive ageing is often required in hybrid QLEDs^16,17^. In 2017, Holloway et al. first reported operando chemical reactions between weak acids and ZnO NPs in QLEDs^18^, and since then, various mechanisms have been proposed to explain these reactions, such as enhanced electron injection via interfacial metallization, surface passivation by acid treatment, and metal ion diffusion^16,19–21^. These mechanisms are believed to underlie the positive ageing phenomenon. However, a recent study offers a contradictory perspective, suggesting that reduced, rather than enhanced, electron injection contributes to improved efficiency^22^. These findings fail to identify whether *n* or *μ* plays a key role in determining *σ* and thus controls the positive ageing effect.

In this study, we investigated the operando physical and chemical properties of ZnO NP films subjected to acid treatment by considering device structure factors and chemical reactions in QLEDs. We demonstrate that acid treatment triggers recrystallization of ZnO NPs, resulting in the formation of a highly efficient electron transport system. This process, initiated by the diffusion of metal ions (Al and Ag), results in the coalescence of ZnO NPs and passivates surface defects. The reduced charge carrier density and improved electron mobility of the recrystallized ZnO NP film not only ensure efficient electron injection into the QDs for exciton formation but also significantly suppress emission quenching. As a result, the EQE of the red QLED increases from 17.2% to 33.7%, representing the highest efficiency reported to date for conventional QLEDs without any light out-coupling structure.

## Results

### Positive-ageing effect in QLEDs

**Fig. 1:**
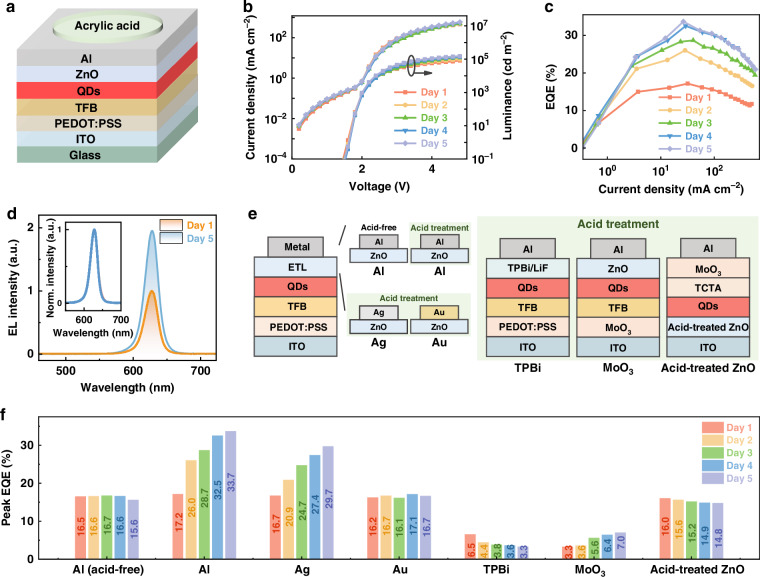
**Positive-ageing effect of QLEDs. a** Device structure of the red QLED. **b** *J‒V‒L* and **c** *EQE‒J* characteristics of the aged device stored for 1–5 days. **d** EL spectra of the device before and after aging, driven at 3 V. The insert depicts the normalized EL spectra. **e** Schematic diagram of ZnO-based QLEDs with different metal cathodes (Al, Ag, and Au), labeled as devices Al (acid-free), Al, Ag, and Au, respectively; TPBi-based QLEDs, labeled as device TPBi; MoO_3_-based QLEDs, labeled as device MoO_3_; and inverted QLEDs with acid-treated ZnO, labeled as device acid-treated ZnO. **f** EQE variation of these devices during storage ageing

To investigate the factors influencing the electron transport behavior of ZnO films and the positive ageing effect, red QLEDs were fabricated with the structure schematically illustrated in Fig. 1a. The temporal evolution of the current density‒voltage‒luminance (*J*‒*V*‒*L*) characteristics of the QLEDs with an Al cathode during acrylic acid treatment is shown in Fig. 1b, with the treatment process detailed in the Methods section. Over time, the device exhibited increased current density, indicating enhanced charge injection and transport, and significantly improved luminance, suggesting more efficient radiative recombination. Notably, the enhancement in radiative recombination is more pronounced than that in charge transport. As shown in Fig. S1c, after five days of ageing, the current density at 5 V increased by approximately 30%, whereas the luminance increased by more than 84%. Consequently, the EQE of the QLED significantly improved from 17.2% in the as-prepared device to 33.7% after five days of ageing, as presented in Fig. 1c. Further ageing the device has little or negative effect on the device performance, likely due to the complete passivation of the ZnO film via Al diffusion under acidic conditions, as discussed later. To the best of our knowledge, the ultimate EQE of 33.7% represents the highest reported value for red QLEDs without light out-coupling structures (Table S1). The electroluminescence (EL) spectra in Fig. 1d confirm consistent and pure emission before and after ageing, without parasitic contributions from adjacent charge transport layers or trap-related recombination. This indicates that the exciton formation zone remains unchanged, with excitons predominantly generated within the quantum dots throughout the ageing process. Additionally, the increased EL intensity at 3.0 V after five days of ageing further supports the enhancement of radiative recombination.

To elucidate the origin of this phenomenon, QLEDs comprising different chemically active metal cathodes (Al, Ag, and Au), a metal oxide hole injection layer, and an organic electron transport layer (ETL) were fabricated with and without acid treatment, as illustrated in Fig. 1e. The evolution of the EQE over the ageing period is summarized in Fig. 1f. Efficiency enhancement is observed exclusively in acid-treated ZnO-based QLEDs employing highly chemically active Al or Ag cathodes in direct contact with the ZnO ETL, irrespective of the hole-injection/transport layers. In contrast, when ZnO is replaced with 1,3,5-tris(N-phenylbenzimidazole-2-yl)benzene (TPBi), the EQE continuously decreases over time. Similarly, inverted QLEDs with acid-treated ZnO ETLs do not exhibit positive ageing effects. Notably, for all the QLEDs, the EL spectra remain unchanged before and after ageing (Fig. S2), indicating that the exciton formation zone is unaffected despite variations in the cathodes and charge transport layers. These findings demonstrate that both the presence of chemically active metals in contact with ZnO and acidic conditions are essential prerequisites for positive ageing in QLEDs, with minimal influence from other layers.

Previous studies suggest that the increase in current density upon ageing (Fig. 1b and Fig. S1a) may result from improved electron injection at the Al/ZnO interface^19^. However, the greater increase in luminance relative to current density (Fig. 1b and Fig. S1b) indicates a significant increase in radiative recombination within the device. This enhancement cannot be attributed solely to improved electron injection into the QDs but originates from fundamental changes in electron dynamics within the ZnO layer due to metal ion diffusion under acidic conditions, i.e., operando chemical reactions. Despite these critical changes, the microscopic structural and electronic modifications in ZnO films during this process have been largely overlooked in previous studies^16–21^.

### Electron transport characteristics of the ZnO NP films

**Fig. 2:**
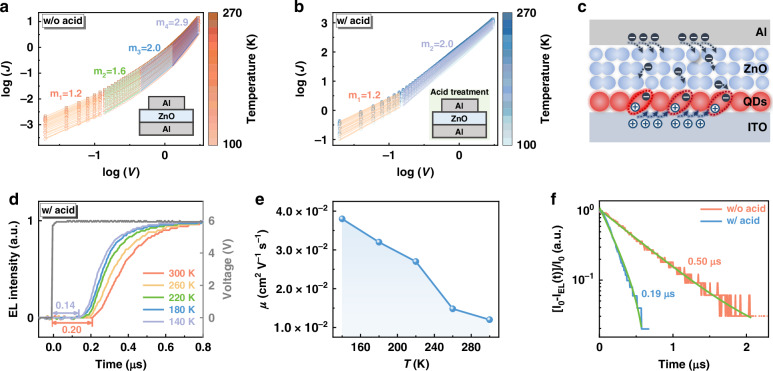
**Electron transport properties of the ZnO NP films. a**, **b** *J‒V* characteristics of Al/ZnO/Al devices (**a**) without acid treatment and (**b**) with acid treatment at different temperatures. The symbols represent the experimental results, with colors corresponding to the measurement temperatures. **c** A schematic diagram of the device structure ITO/QDs/ZnO/Al and its working mechanism for measuring electron mobility in ZnO. **d** TrEL spectra of ITO/QD/ZnO/Al devices at different temperatures. **e** Temperature-dependent mobility of a ZnO film with acid treatment. **f** Plot of $\left[{I}_{0}-{I}_{{EL}}\left(t\right)\right]/{I}_{0}$ as a function of time, with the green line representing the fitted curve

Given the dramatic increase in device luminance shown in Fig. 1b, the electron transport behavior of the acid-treated ZnO film is expected to differ significantly from that of the initial as-prepared ZnO NP film. To eliminate the influence of charge balance and recombination on current drift‒diffusion processes, single-carrier devices were employed to simulate the electron injection and transport properties of charge carriers in the ZnO films. Electron-only devices (EODs), consisting of glass/Al (~70 nm)/ZnO (~120 nm)/Al (~100 nm), were prepared, and the ZnO layer thickness was confirmed by AFM measurements (Fig. S3). The temperature-dependent *J* − *V* curves for the control (without acid treatment) and acid-treated EODs are shown in Fig. 2a, b. The current‒voltage characteristics follow a power-law dependence ($J\propto {V}^{m}$), with distinct transport regimes identified through exponent variations^23,24^. The control ZnO device exhibited four regions characterized by m = 1.2 (ohmic conduction), 1.6 and 2.0 (shallow-trap SCLC), and 2.9 (deep-trap SCLC). In contrast, acid-treated devices demonstrate simplified transport behavior with only two regimes: m = 1.2 (ohmic) and m = 2.0 (trap-free SCLC). This marked contrast in scaling exponents indicates effective trap state passivation through acid treatment, significantly reducing both shallow and deep trap densities in ZnO compared with those in pristine films. Notably, by comparing the *J* − *V* curves in the ohmic region shown in Fig. 2a, b, the device with acid treatment exhibited a higher current density than the control, indicating a significant increase in the conductivity of the ZnO films. Typically, passivation of defect states leads to a reduction in carrier concentration, which generally results in a decrease in conductivity. According to the relationship *σ* = *neμ*, conductivity depends not only on carrier concentration but also significantly on carrier mobility.

To elucidate the contribution of electron mobility to the conductivity, the electron mobility of the ZnO layers was measured via the time-resolved electroluminescence (TrEL) technique^25^. Devices without a hole-transport layer were fabricated, as depicted in Fig. 2c, with both the QD and ZnO layer thicknesses determined by AFM (Fig. S4). The exciton formation zone is mainly localized at the QD/ITO interface due to the poor hole mobility in the QD layer^26^. Pure red emission (Fig. S5) without any parasitic contribution from ZnO indicates that excitons are dominantly formed in the QDs, independent of the temperature. Therefore, the delay time (*t*_*d*_) of the TrEL response to the driving electrical pulse is primarily determined by electron transport through the ZnO layer. As shown in Fig. S6, *t*_*d*_ increases with decreasing temperature in the device without acid treatment, indicating a reduction in mobility. This behavior suggests that electron transport in ZnO NPs is consistent with the hopping model^27,28^, where lower temperatures reduce hopping probabilities between localized states. In contrast, as shown in Fig. 2d, *t*_*d*_ decreases and mobility increases for the acid-treated device as the temperature decreases, suggesting that a band-like transport model may be induced, where reduced lattice vibrations at lower temperatures decrease phonon scattering. According to our recently reported method^25^, the electron mobility with temperature for the acid-treated ZnO film was calculated and is plotted in Fig. 2e. Additionally, the electron mobility of the acid-treated ZnO film was calculated to be approximately 1.2 × 10^−2^ cm^2^ V^−1^ s^−1^, three orders of magnitude greater than that of the as-prepared ZnO film (see Fig. S7 and Table S2), indicating that the enhanced conductivity should be attributed to increased mobility.

The charge injection dynamics were further assessed by examining the rising edges of the TrEL response for these hole-transport layer-free devices (Fig. S7a). Owing to the limited hole transport (Fig. 2c), the EL onset is primarily determined by electron injection and transport. The rising edges are replotted in Fig. 2f according to the formula $I\left(t\right)={[I}_{0}-{I}_{{EL}}(t)]/{I}_{0}$, where ${I}_{{EL}}\left(t\right)$ is the EL intensity at time *t* and where ${I}_{0}$ is the saturated EL intensity^29^. The EL decay follows a single exponential, which is consistent with electron injection and transport being dominant in this device. The electron injection rate for the acid-treated device was 5.26 × 10^6^ s^−1^, approximately double that of the control device (2.0 × 10^6^ s^−1^). These results further suggest that efficient electron transport is readily achieved in the acid-treated ZnO layer.

Hall measurements were also performed to evaluate the electron mobility of the ZnO films. The average mobility of the acid-treated ZnO films is 1.44 × 10^3^ cm^2^ V^−1^ s^−1^ (see Fig. S8a), which is significantly greater than that of the untreated ZnO films (1.75 × 10^2^ cm^2^ V^−1^ s^−1^). This high mobility is comparable to previous reports on QDs and organic semiconductors exhibiting band-like transport^30,31^, further confirming the possibility of band-like transport in acid-treated ZnO films. Additionally, the reduction in carrier concentration from 1.54 × 10^14^ cm^−3^ in the untreated ZnO film to 2.03 × 10^13^ cm^−3^ in the acid-treated film (Fig. S8b) confirms that defect passivation occurred during the ageing process. This suggests that the observed conductivity enhancement is due primarily to the increase in mobility rather than to changes in carrier concentration. Notably, the significant disparity in electron mobility values obtained from the TrEL and Hall measurements is due to the distinct principles underlying these techniques. Owing to the similar driving modes of the LEDs and the TrEL, the mobility measured by the TrEL accurately reflects the intrinsic charge transport properties of an operating device. Therefore, we utilized the mobility values from the TrEL measurements to analyze the EL performance.

### Recrystallization mechanism of the ZnO NPs

The passivation effects of metal ions and acid on the ZnO NPs were evaluated by measuring the photoluminescence (PL) spectra of the ZnO and ZnO/Al films, both with and without acid treatment (Fig. S9). ZnO (~60 nm) single layers and ZnO (~60 nm)/Al (~70 nm) bilayers were deposited on Si/SiO_2_ (200 nm) substrates, with the Al layers removed via 3 M tape before PL measurement to avoid interference with the ZnO emission. The emission peak at 357 nm corresponds to the intrinsic excitonic emission of the ZnO NPs, whereas the peak at approximately 545 nm is attributed to V_O_-related emissions^32,33^. All the data were normalized to the excitonic emission to highlight changes in the defects on the surface of the ZnO NPs. Notably, the defect-related emission of the ZnO film significantly decreases after Al deposition, suggesting passivation of the ZnO NPs by the Al ions. Furthermore, acid treatment further reduces this emission, implying enhanced passivation of V_O_-related defects, as shown in Fig. 2. These findings suggest that the surface states of ZnO NPs are highly susceptible to metal ions and acid treatment.

**Fig. 3:**
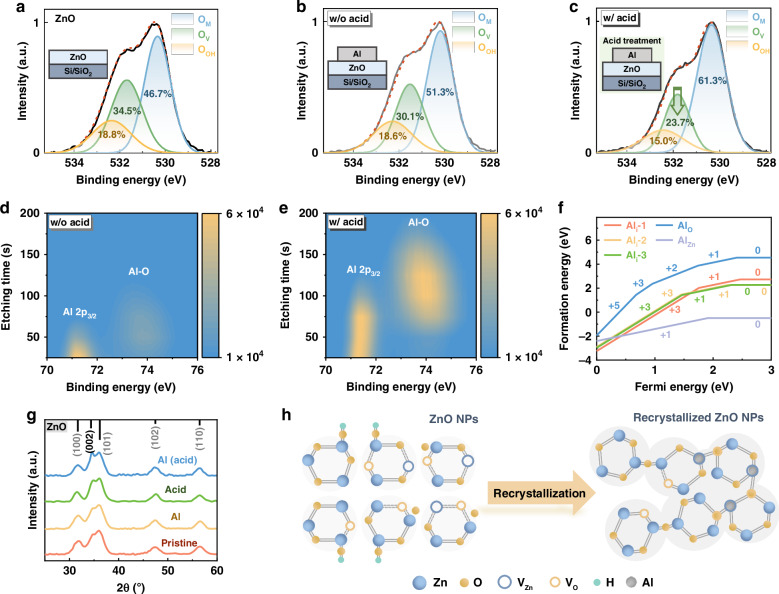
**Operando chemical reactions of ZnO NPs. a**–**c** Deconvolution of the O 1 s XPS peaks of the **a** pristine ZnO film. **b** Al-deposited ZnO film without acid treatment and **c** Al-deposited ZnO film with acid treatment. **d**, **e** Evolution of the Al 2p XPS peaks of the Al-deposited ZnO films (**d**) without acid treatment and (**e**) with acid treatment as a function of the Ar ion etching time. **f** Formation energies of various defects in ZnO films with Al doping. **g** XRD patterns of four ZnO samples: pristine ZnO, Al-deposited ZnO film (Al), ZnO film with acid treatment (acid), and Al-deposited ZnO film with acid treatment (Al (acid)). **h** Schematic illustration of the recrystallization process of ZnO nanoparticles

X-ray photoemission spectroscopy (XPS) spectra for pristine ZnO, ZnO/Al, and acid-treated ZnO/Al films (Fig. 3a–c) show that the O 1 s signal decomposes into three Gaussian peaks representing metal‒oxygen bonds (O_M_), V_O_, and hydroxyl groups (OH) at binding energies of 530.3, 531.7, and 532.4 eV, respectively^33,34^. After Al deposition, the proportion of V_O_ defects decreases from 34.5% to 30.1%, and acid treatment further reduces this percentage to 23.7%, confirming that Al ion diffusion effectively passivates V_O_ defects, especially under acidic conditions.

XPS depth profiling further verified that Al diffused into ZnO (Fig. S10a). The samples were prepared similarly to those used for the PL measurements, with the Al films removed prior to analysis. Owing to challenges in determining the ZnO thickness during ion beam etching, the core-level spectra of Al, Zn, and O were tracked as a function of etching time. To mitigate air exposure effects, the XPS spectra at 0 s of etching were omitted. As shown in Fig. 3d (and Fig. S10b), the intensity of the Al-2p_3/2_ peak (~71.2 eV) decreases along the depth profile, indicating a reduced gradient distribution of Al ions within the ZnO layer. The formation of Al‒O bonds (~73.8 eV) suggests an operando chemical reaction during Al diffusion, which is consistent with previous reports^16,19^. For the sample without acid treatment, the Al-O and Al 2p_3/2_ signals disappear after 125 s of etching, indicating limited Al diffusion. In contrast, the acid-treated samples exhibit strong Al‒O signals persisting beyond 200 s of etching, as shown in Fig. 3e (and Fig. S10c), indicating more efficient diffusion and operando chemical reactions of the Al ions in the ZnO layer under acidic conditions. The emergence of Si core peaks at 200 s of etching (Fig. S10d) confirms that Al ions permeate the entire ZnO layer in the acid-treated samples. For the Au-capped ZnO layers, the Au signals are confined to a few nanometers near the ZnO film surface (Fig. S10e, f), with no discernible acid-induced diffusion effects. These findings suggest that the diffusion of highly chemically active metal ions is facilitated under acidic conditions, playing a crucial role in the positive ageing effect of ZnO-based hybrid QLEDs.

Fourier transform infrared (FTIR) spectroscopy was conducted to analyze bond formation during operando chemical reactions (Figs. S11 and S12). Al-O-Zn absorption peaks at 649, 723, and 808 cm^−1^ (Fig. S12b, c) are observed in the ZnO/Al samples with and without acid treatment, confirming that Al is incorporated into the ZnO NP crystal lattice. Furthermore, Al-O-Zn signals were detected at depths of approximately 49 nm in the acid-treated samples, which is consistent with the XPS results. Additionally, the Zn 2p peaks shift toward lower binding energies in the ZnO/Al films (Fig. S13), with a more pronounced shift after acid treatment, indicating that Al is incorporated into the zinc vacancies (V_Zn_). To further explore the Al occupation of Zn sites, theoretical calculations were performed to evaluate the formation energies of Al interstitial defects (Al_i_-1, Al_i_-2, and Al_i_-3), Al substitutional defects at O sites (Al_O_), and Al substitutional defects at Zn sites (Al_Zn_). As shown in Fig. 3f, Al_Zn_ has a notably negative formation energy, indicating that Al atoms readily substitute for Zn and form Al‒O bonds, which is consistent with the experimental findings.

The influence of metal ion diffusion under acidic conditions on the crystallinity of ZnO nanoparticles (NPs) was examined via X-ray diffraction (XRD) analysis (Fig. 3g). All the samples exhibit characteristic peaks corresponding to the hexagonal wurtzite ZnO structure^35,36^, indicating that Al diffusion does not alter the crystal structure. The ZnO NPs synthesized through the hydrolysis of zinc acetate exhibit a high density of hydroxide and acetate groups and absorb oxygen on their surface^37,38^. The Al ions should primarily passivate the V_O_ and V_Zn_ defects by reacting with the absorbed oxygen and various surface groups instead of forming AlO_x_ (if any, they are very few). Further analysis revealed that, in the acid-treated ZnO/Al bilayer, the (002) diffraction peak was discernibly enhanced and sharpened, as confirmed by the high consistency of measurements across multiple tests shown in Fig. S14. This indicates the coalescence of ZnO NPs along the *c* axis^39^, referred to as recrystallization of the ZnO NPs in the solid film. In addition, the narrowing of the (110) peak in the XRD pattern suggests an increase in the size of the ZnO nanoparticles, as illustrated in Fig. S15.

The recrystallization of ZnO NPs is schematically illustrated in Fig. 3h. Two possible recrystallization mechanisms are proposed. On the one hand, metal ions diffuse into the ZnO layer, with Al occupying the V_Zn_ in the lattice and stabilizing surface-adsorbed oxygen, forming Al‒O‒Zn bonds and promoting ZnO NP aggregation. On the other hand, dehydration reactions between hydroxyl groups within ZnO NPs result in aggregation via Zn‒O‒Zn bonds. The hydroxyl groups predominantly originate from the synthesis process and acid-assisted surface oxygen stabilization under acidic conditions^10^. The decrease in the OH^−^ group proportion (from 18.8% in the initial ZnO film to 18.6% and 15.0% after Al deposition and acid treatment), as shown in Fig. 3b, c, indicates that dehydration reactions occur within the ZnO NPs. Additional evidence for these operando dehydration reactions is the detection of water. The ^1^H liquid-state nuclear magnetic resonance (NMR) spectra of various ZnO samples (Fig. S16) revealed an increase in the water signal at 3.33 ppm after Al and acid treatment (Fig. S17), confirming the occurrence of operando dehydration reactions in the ZnO films. On the basis of the above results, a mechanism for the operando chemical reactions in the ZnO films can be proposed. During the spin-coating process, ZnO NPs aggregate randomly in the films. The wurtzite structure of the ZnO NPs has different reactivities for the (002) planes at the bottom and top of the *c* axis. Metal ions fill and passivate related defect states, whereas organic acids induce dehydration reactions between hydroxyl groups, resulting in the formation of Al-O or Zn-O bonds and ultimately promoting the recrystallization of ZnO into long-range nanorod-like structures.

**Fig. 4:**
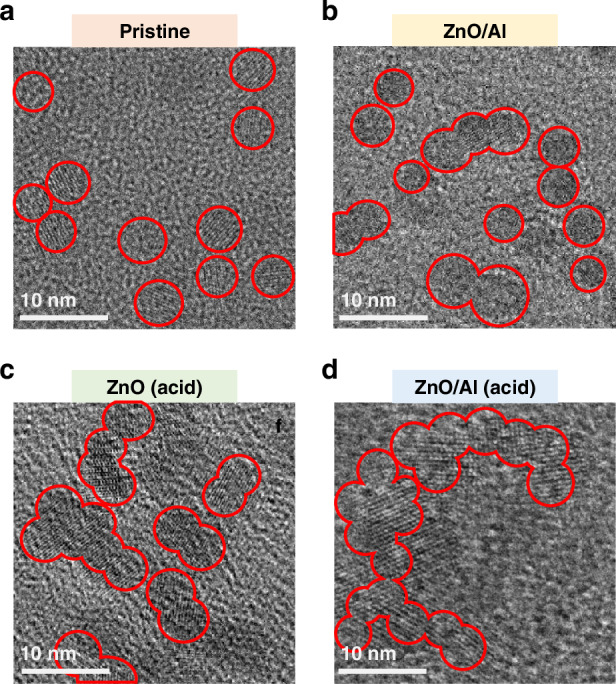
**Morphology of the ZnO NPs. a**–**d** TEM images of **a** pristine ZnO, **b** Al-deposited ZnO, **c** ZnO with acid treatment, and **d** Al-deposited ZnO with acid treatment

To further verify the above proposal, transmission electron microscopy (TEM) was performed on the ZnO NPs extracted from various samples. The pristine ZnO film without Al or acid treatment had the best dispersibility (Fig. S18). In contrast, films treated with Al or acid show significant sedimentation (insoluble larger ZnO crystals) at the bottom of the EP tube. TEM analysis of the supernatant revealed that the ZnO NPs from the pristine film exhibited excellent dispersity, with an average diameter of approximately 5.2 nm (Fig. 4a). In contrast, the ZnO NPs coalesced when the film was treated with Al or acid (Fig. 4b, c). Coalescence is further enhanced when both acid and Al ions coexist, forming long-range ZnO nanocrystals (Fig. 4d), confirming that recrystallization is most effective under these conditions. High-resolution TEM (HRTEM) images (Fig. S19) clearly show continuous crystal lattice fringes, confirming the formation of long-range ZnO nanocrystals rather than the conventional agglomeration of neighboring NPs. Multiple independent TEM tests further verified these findings, yielding consistent results (Fig. S20). The (002) crystal orientation of the coalesced ZnO NPs further supports the formation of long-range ZnO nanocrystals, which is consistent with the XRD results in Fig. 3g. In contrast, the ZnO NPs from the Au-covered samples remained well dispersed in ethanol (Fig. S21), further supporting the hypothesis that high-chemical-activity metals are prerequisites for ZnO NP recrystallization.

## Discussion

**Fig. 5:**
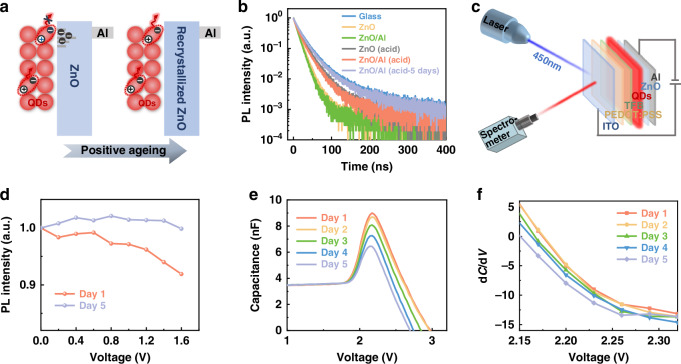
**Charge–carrier dynamics in the QDs. a** Schematic illustration depicting exciton quenching of QDs by ZnO before and after recrystallization. **b** PL decay curves measured for the QDs under different conditions: QDs on glass, denoted as glass; glass/QDs/ZnO, denoted as ZnO; glass/QDs/ZnO/Al, denoted as ZnO/Al; glass/QDs/ZnO with acid treatment, denoted as ZnO (acid); glass/QDs/ZnO/Al with acid treatment, denoted as ZnO/Al (acid); and glass/QDs/ZnO/Al with acid treatment after 5 days of aging, denoted as ZnO/Al (acid-5 days). **c** Schematic diagram of the setup for PL spectra testing at a voltage below the turn-on voltage for electroluminescence. **d** PL intensity versus voltage curves of the device before and after aging. **e**, Capacitance‒voltage curves of the device during positive aging, and **f**, the derivative of capacitance (d*C/*d*V*) as a function of voltage (*V*) during positive aging

The influence of the recrystallized ZnO film on the emission of the QD emitters was examined by revisiting the exciton dynamics in the optical and electrical excitation regimes. Passivation of the ZnO NPs effectively protects the excitons in the QDs from quenching by the defects, as illustrated schematically in Fig. 5a. The higher conduction energy level of ZnO suppresses charge separation (i.e., emission quenching) in the QDs by passivating/eliminating defects on the surface of the ZnO NPs. The PL decays of the QDs under different conditions were measured, and the results are shown in Fig. 5b and summarized in Table S3. The average PL lifetime of the QDs on the inert glass substrate is approximately 24.10 ns, which decreases to 12.62 ns when the ZnO layer is deposited. This reduction is attributed to the quenching effect induced by the ZnO NPs, as previously reported^40,41^. The PL decay of the QDs is further accelerated when a ~100 nm thick aluminum film is deposited on the ZnO layer, possibly because of the localized field effect caused by the highly reflective metal film. Upon acid treatment, the PL decay of the QDs gradually recovers, reaching 23.26 ns after 5 days of ageing, nearly identical to that on the inert glass substrate. These results further demonstrate the effective passivation of ZnO NPs through Al-ion diffusion under acidic conditions. Notably, the device maintains an identical Lambertian distribution during the ageing period (Fig. S22), indicating that metal penetration does not affect the optical properties of the devices.

For the influence of charge carriers on the emission of QDs, the PL emission of the QD emission layer in QLEDs under a low electric field is recorded, as shown schematically in Fig. 5c. When the voltage is below 1.6 V, the device is not turned on, meaning that only a few electrons are injected into the QDs. In Fig. 5d, for the as-prepared QLED, the PL emission monotonically decreases as the applied voltage increases because of the accumulation of electrons within the defect-enriched ZnO layer. In contrast, the recrystallized ZnO layer, which possesses very few defects, suppresses electron accumulation, resulting in a stable PL intensity of up to 1.6 V for the aged QLED. The decrease in peak capacitance with ageing, as shown in Fig. 5e, further confirms the reduction in trapped electrons within the device. Notably, the decrease in capacitance after the peak accelerated with increasing ageing time (Fig. 5f), indicating that the charge carrier consumption increased in the aged device. This can be attributed to the highly efficient electron transport behavior, where increased electron mobility leads to sufficient and fast enough electron injection into the QDs with little trapping in the ZnO, approaching the limiting condition that the electrons for forming excitons can be taken as desired for the QDs. As a result, accelerated recombination, rather than charge trapping, governs the charge dynamics in aged QLEDs. Furthermore, the operational stability of the aged device is significantly improved. As shown in Fig. S23, the *T*_50_ (time for luminance to decrease to 50% of the initial value) of the aged device at an initial luminance of approximately 10,000 cd m^−2^ increases from 9.04 h to 14.49 h, corresponding to an extrapolated *T*_50_ of 57,686 h at 100 cd m^−2^.

In summary, we have demonstrated the operando recrystallization effect within the ZnO NP layer, a widely utilized ETL in various photoelectric devices based on QDs, perovskites, and small molecule/polymer organic materials. This novel finding, which has not been previously reported, results in the formation of highly defect-passivated and long-range ZnO nanocrystals. The recrystallization process shifts the transport mechanism from hopping to potential band-like electron conduction in the ZnO film. As a result, electron injection into the QLEDs is sufficient in an “injection-on-demand” manner, which helps establish a dynamic charge balance and leads to relatively high device efficiency. Our results further suggest that, owing to the surface effects of nanocrystal materials, operando chemical reactions at various interfaces and functional layers in QLEDs require special attention. This study demonstrates that operando engineering of nanocrystals with highly chemically active surfaces offers a promising pathway for tuning their photoelectrical properties, ultimately enabling the realization of highly efficient devices through a post-treatment process.

## Methods

### Materials

Poly(3,4-ethylenedioxythiophene) polystyrene sulfonate (PEDOT:PSS Clevios P VP AI 4083) aqueous solution, poly(9,9-dioctylfluoreneyl-2,7-diyl)-alt-(4,4’-(N-(4-butylphenyl) (TFB), and 1,3,5-tris(N-phenylbenzimidazol-2-yl) benzene (TPBi) were obtained from Xi′an Yuri Solar Co., Ltd., and 4,4’,4-tris(carbazol-9-yl) triphenylamine (TCTA) and MoO_3_ were purchased from Luminescence Co. LiF and QDs were purchased from Aladdin and Guangdong Poly OptoElectronics Co., Ltd. ZnO nanoparticles (NPs), synthesized according to our previous report^42^, were dissolved in ethanol at a concentration of 40 mg mL^−1^.

### Device fabrication

QLEDs consisting of glass/ITO (~150 nm)/PEDOT:PSS (~35 nm)/TFB (~30 nm)/QDs (~23 nm)/ZnO (~50 nm)/metal cathodes (~100 nm) were fabricated. Prior to device fabrication, the ITO substrates were ultrasonically cleaned with acetone, ethanol, and deionized water in sequence for 15 min each. These substrates were subsequently blow-dried with N_2_ flow, followed by UV-ozone treatment for 9 min to enhance surface wettability. The PEDOT:PSS aqueous solution, filtered through a 0.45 µm filter, was spin-coated at a speed of 4000 rpm on the substrates and annealed at 120 °C for 30 min before being transferred to a nitrogen-filled glovebox. Films of TFB, QDs, and ZnO were fabricated as described in our previous reports^43^. Finally, the Al, Ag, and Au electrodes were thermally evaporated in a high-vacuum chamber with a base pressure below 4.5 × 10^−5^ Pa. The QLED devices were placed in a custom-built airtight box (the volume of the box was approximately 20 cm^3^) containing 10 µL of acrylic acid. They were stored in a glovebox, retrieved daily for measurements, and then returned.

### Characterizations of QLEDs

The current *density‒voltage‒luminance (J‒V‒L*) characteristics of the QLEDs were measured via a programmable power supply (Keithley 2400) combined with a luminance meter (Minolta LS-110). Electroluminescence (EL) and photoluminescence (PL) spectra were recorded by an Ocean Optics Maya 2000 Pro spectrometer. An excitation laser source with a peak wavelength of 420 nm was used for the PL measurements. The PL decay of the QDs (excited at 402 nm) was acquired via a fluorescence spectrometer (QuantaMaster 8000). The transient electroluminescence (TrEL) response was measured via a custom-built system as described in detail in previous publications^25^. The capacitive characteristics of the devices were recorded by a precision LCR meter (Tonghui TH2829C) at a frequency of 1 kHz and an AC voltage of 100 mV. The atomic force microscopy (AFM) measurements were performed via a Core-AFM from Nanosurf. Temperature-dependent tests were conducted with a Lake Shore CRYOTRONICS cryogenic platform system.

### Characterization of ZnO NPs

The PL spectra of the ZnO NPs were acquired with 320 nm laser excitation. XPS measurements were performed via a Thermo Scientific NEXSA X-ray photoelectron spectrometer, with peak fitting performed via XPSPEAK and a Gaussian‒Lorentzian function with Shirley background subtraction applied. X-ray diffraction (XRD) patterns of the ZnO films were acquired via a Rigaku SmartLab SE X-ray diffractometer from the Instrument and Equipment Sharing Platform of the College of Physics, Jilin University. Fourier transform infrared (FT-IR) spectra were measured via a Shimadzu Affinity-1 FT-IR spectrometer. Transmission electron microscopy (TEM) and high-resolution TEM (HRTEM) images of the ZnO nanocrystals were captured via a JEOL JEM-2200FS instrument. Hall effect measurements were conducted via the 775HMS Matrix Hall Effect Testing System from Lakeshore. For the Hall effect measurements, the samples were either subjected to a 5-day acid treatment or left untreated after depositing ZnO (~200 nm thick) and Al films on the substrates. Prior to the measurements, the Al electrodes were carefully removed. The point defects of Al were calculated via density functional theory (DFT) via the Perdew–Burke–Ernzerhof (PBE) functional, with calculations performed via the Vienna ab initio Simulation Package (VASP)^44,45^. The formation energy was calculated via the following formula:

$$\Delta {H}_{\alpha ,q}\left({E}_{F},\mu \right)={E}_{\alpha ,q}-{E}_{H}-\sum _{i}{n}_{i}{\mu }_{i}+q\left({E}_{F}+{E}_{V}+\Delta V\right)+{E}_{{corr}}$$

where ${E}_{\alpha ,q}$ and *E*_*H*_ are the total energy derived from a supercell calculation with a defect in a charged state q and the total energy of the perfect supercell, respectively, and where *n*_*i*_ represents the number of atoms that have been added to (*n*_*i*_ > 0) or removed (*n*_*i*_ < 0) from the perfect supercell. *μ*_*i*_ is the chemical potential of the corresponding atom. *E*_*F*_ is the Fermi energy relative to *E*_*V*_ at the top of the valence band of the original unit cell, and ∆*V* is the difference in the reference potentials of the defective and perfect supercells.

## Supplementary information

Supplementary Information for Operando ZnO recrystallization for efficient quantum-dot light-emitting diodes

## Data Availability

All data supporting the findings of this study are included in the paper and its Supplementary information files. Additional data can be obtained from the corresponding author (W.Y.J.) upon reasonable request.
