# Supplementary material for: Operando ZnO recrystallization for efficient quantum-dot light-emitting diodes
Source: Light Sci Appl. 2025 May 15;14:196. doi: 10.1038/s41377-025-01867-1 (PMC12078497; doi:10.1038/s41377-025-01867-1)
Supplement: Supplementary file 1 — Supplementary Information for Operando ZnO recrystallization for efficient quantum-dot light-emitting diodes [file 41377_2025_1867_MOESM1_ESM.pdf]

Supplementary Information for

Operando ZnO recrystallization for efficient quantum-dot light-emitting diodes

Song Wang[1,4], Shihao Liu[2,4], Ting Wang[3], Jialin Bai[1], Jingyu Peng[1], Hanzhuang Zhang[1]✉, Wenfa Xie[2]✉ & Wenyu Ji[1]✉

[1]Key Lab of Physics and Technology for Advanced Batteries (Ministry of Education), College of Physics, Jilin University, Changchun, 130012, China

[2]State Key Laboratory of Integrated Optoelectronics, College of Electronic Science and Engineering, Jilin University, Changchun 130012, China

[3]Key Laboratory of Functional Materials Physics and Chemistry of the Ministry of Education, Jilin Normal University, Changchun 130103, China

[4]The authors contribute equally to this work.

✉e-mail: zhanghz@jlu.edu.cn; xiewf@jlu.edu.cn; jiwy@jlu.edu.cn

Table of Contents:

I. Supplementary figures and tables

Figure S1. Optoelectronic characteristics of the QLED during positive ageing.

Figure S2. Electroluminescence of various devices during positive ageing.

Figure S3. Characterization of ZnO film thickness.

Figure S4. Moophology properties of QD and ZnO films.

Figure S5. Temperature-dependent EL spectra of ITO/QDs/ZnO/Al device with acid treatment.

Figure S6. Temperature-dependent TrEL spectra of the ITO/QDs/ZnO/Al device without acid treatment.

Figure S7. Transient properties of the hole-transport layer-free devices.

Figure S8. Hall effect measurements of ZnO films.

Figure S9. PL spectra of ZnO films.

Figure S10. Metal diffusion under acid treatment.

Figure S11. Sample preparation for the FTIR measurements.

Figure S12. Fourier transformed infrared (FTIR) spectroscopy characterization.

Figure S13. Zn 2p core level XPS spectra.

Figure S14. XRD patterns of four types of ZnO samples from three batches.

Figure S15. XRD patterns of ZnO films at (110) peak.

Figure S16. Nuclear magnetic resonance spectra of four samples.

Figure S17. Intensity of water signal in the four samples.

Figure S18. Redispersion of ZnO film in ethonal.

Figure S19. High-Resolution transmission electron microscopy images.

Figure S20. TEM images of four types of ZnO films from three batches.

Figure S21. TEM image of Au-covered ZnO sample.

Figure S22. Angular distribution of light intensity over 5 days of positive ageing.

Figure S23. Operational stability of the QLEDs.

Table S1. Summaries of device performance for red CdSe-based devices.

Table S2. Summary of the calculated parameters for ZnO thin films in devices.

Table S3. Double-exponential fitting parameters for PL decay of QDs.

II. Supplementary references

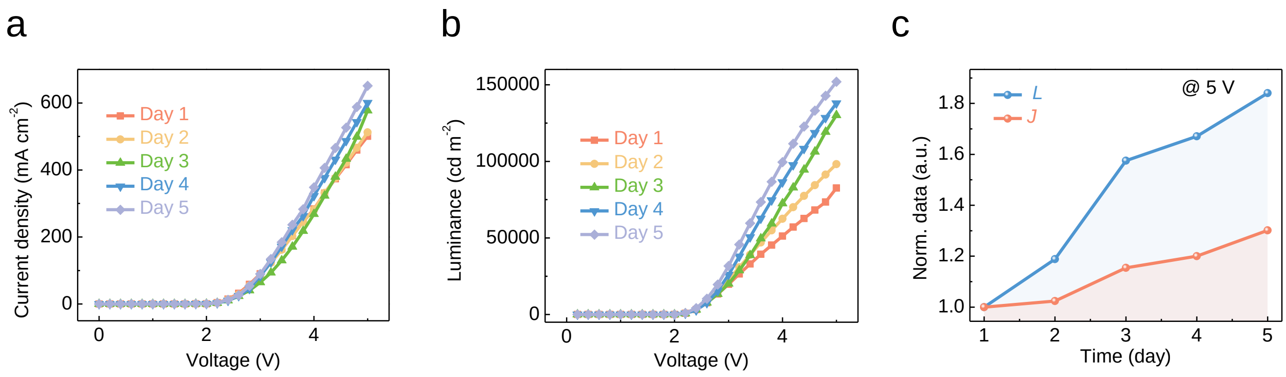

Figure S1. Optoelectronic characteristics of the QLED during positive ageing. a, *J*–*V* and **b**, *L*–*V* curves for the QLED (ITO/PEDOT:PSS/TFB/QDs/ZnO/Al) during ageing under acidic conditions. **c**, Time-dependent curves of current density and luminance at 5 V, normalized to the data from the first day.

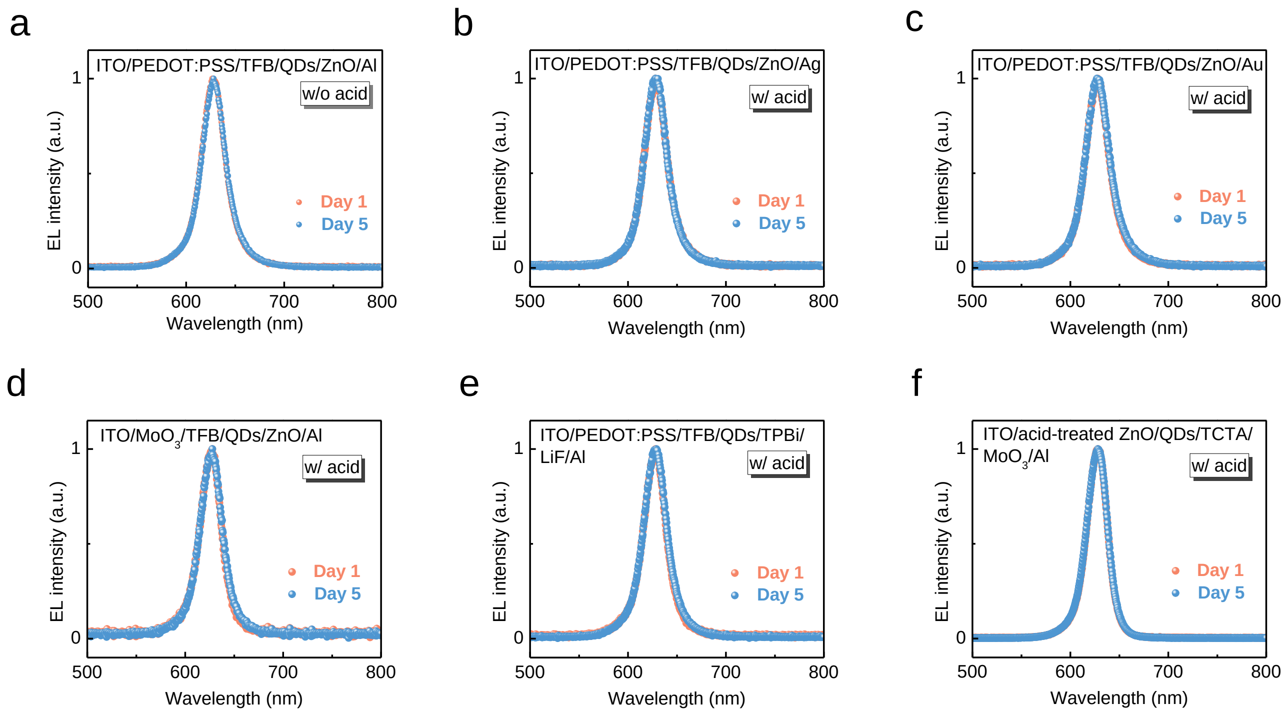

Figure S2. Electroluminescence of various devices during positive ageing. a-f, EL spectra for the as-prepared (Day 1) and the aged for five days (Day 5) QLEDs with different structures.

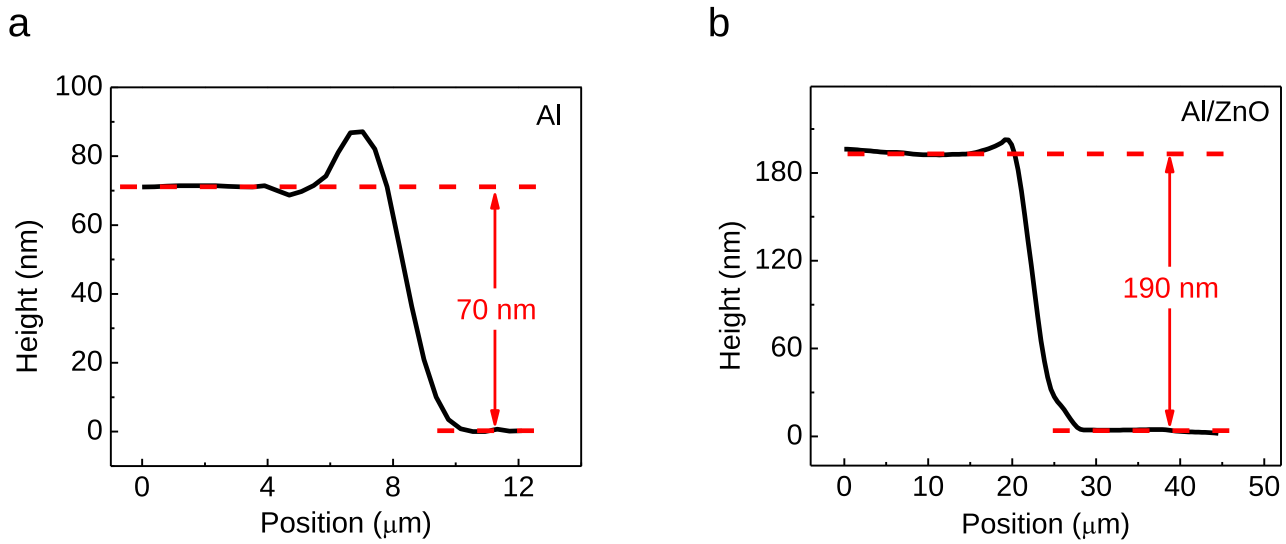

Figure S3. Characterization of ZnO film thickness. Evaluating the thicknesses **a**, Al and **b**, Al/ZnO through AFM measurement. The result reveals that the thickness of ZnO layer is around 120 nm.

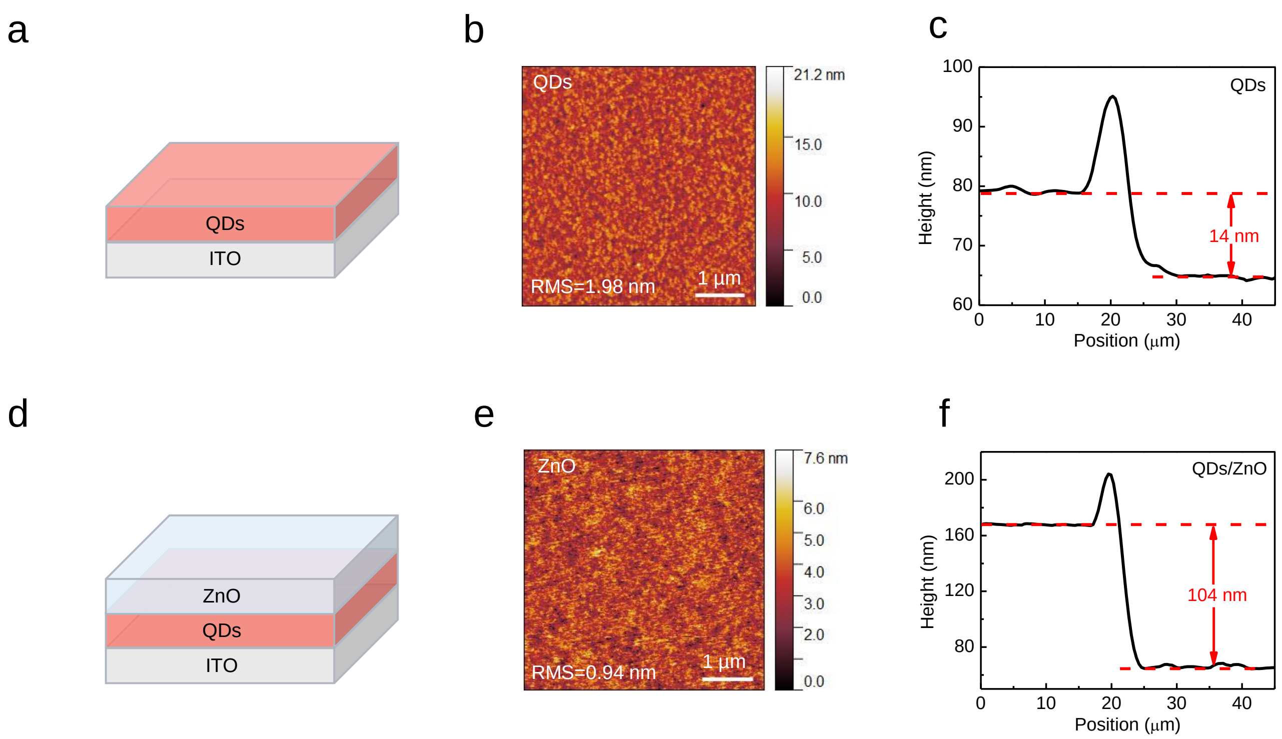

Figure S4. Moophology properties of QD and ZnO films. a, Sample of ITO/QDs. **b**, AFM height morphology and **c**, thickness measurement of the QD film. **d**, Sample of ITO/QDs/ZnO. **e**, AFM height morphology and **f**, thickness measurement of the ITO/QDs/ZnO.

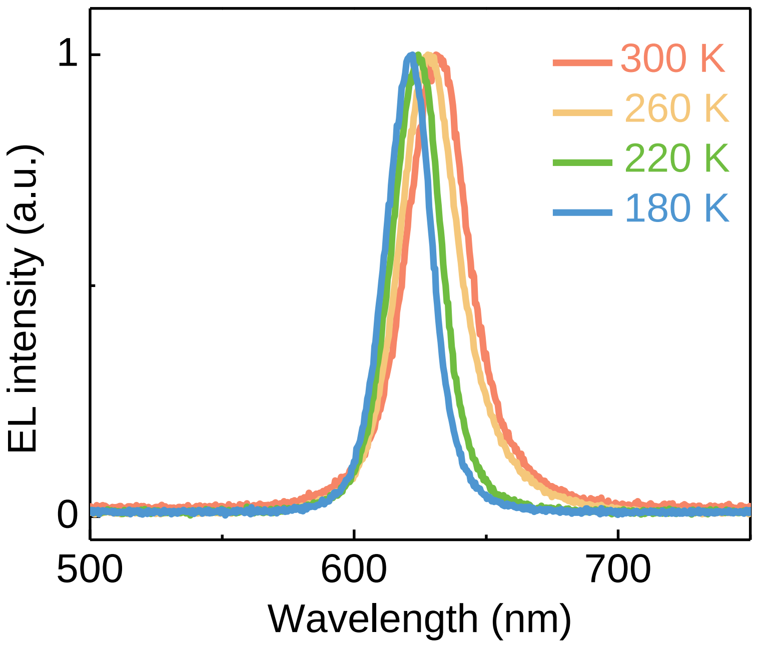

Figure S5. Temperature-dependent EL spectra of ITO/QDs/ZnO/Al device with acid treatment.

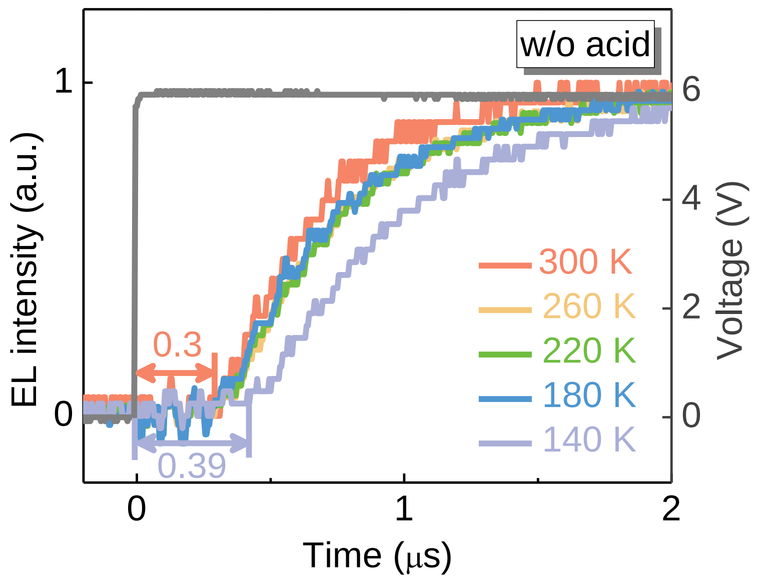

Figure S6. Temperature-dependent TrEL spectra of the ITO/QDs/ZnO/Al device without acid treatment.

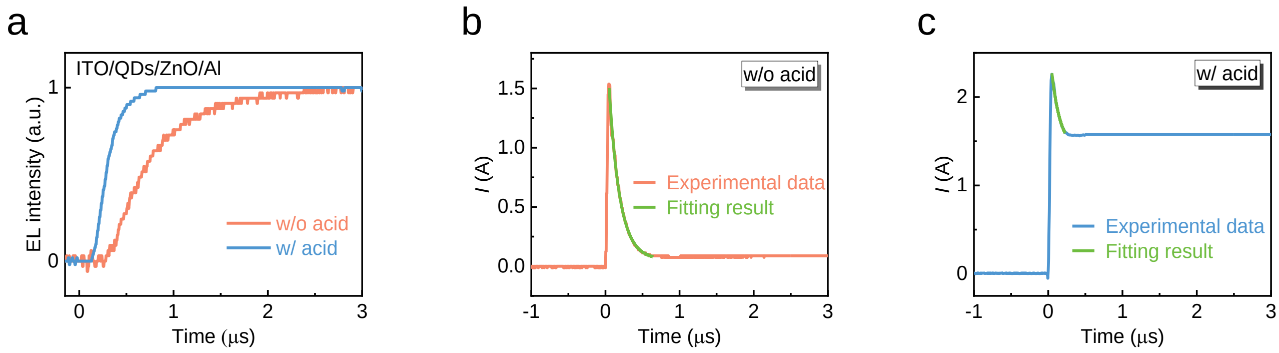

Figure S7. Transient properties of the hole-transport layer-free devices. a, TrEL response of devices at 270 K. Transient currents of devices **b**, without acid treatment and **c** with acid treatment.

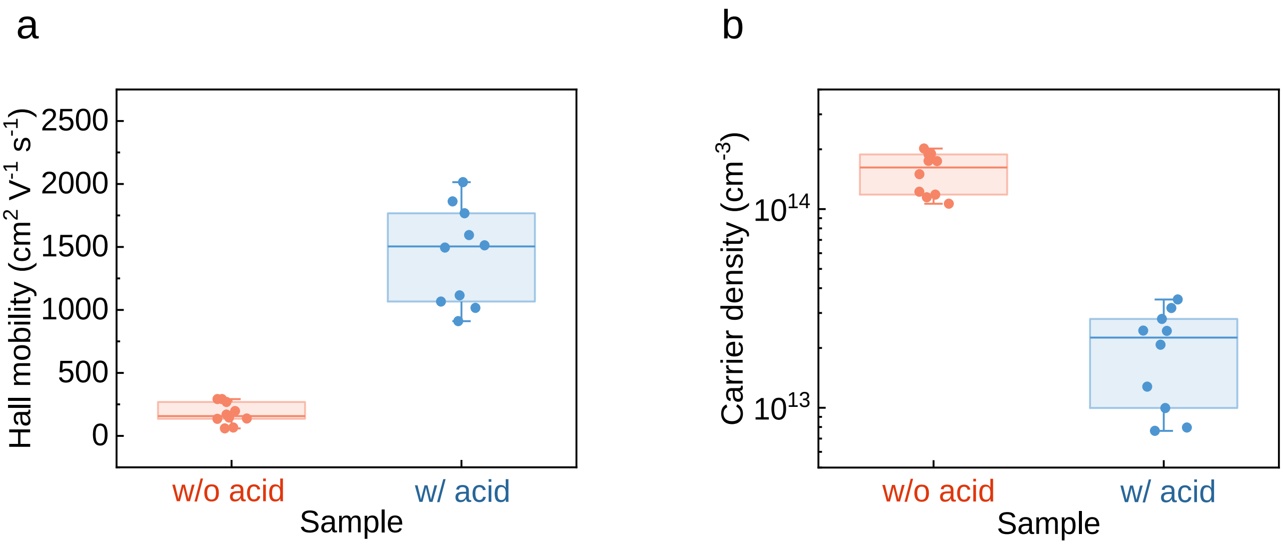

Figure S8. Hall effect measurements of ZnO films. a, Electron mobility and **b**, carrier density of ZnO films with and without acid treatment (~200 nm thick) obtained by Hall measurements.

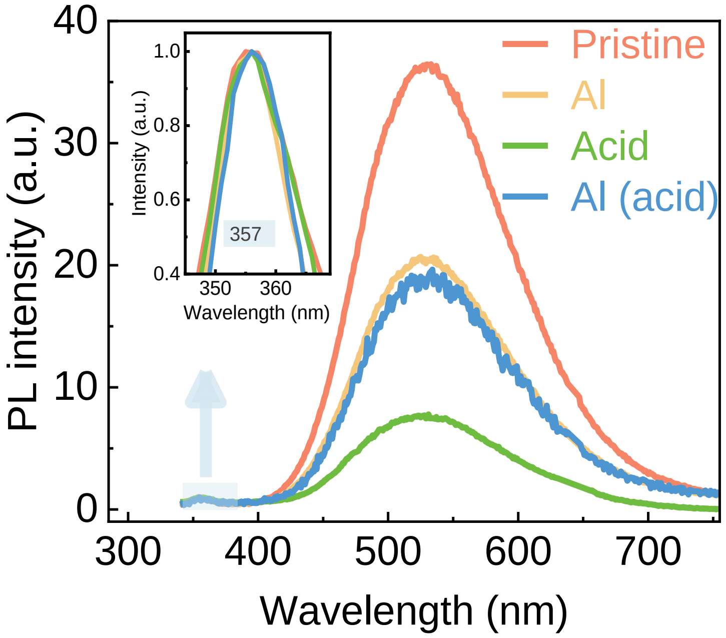

Figure S9. PL spectra of ZnO films.

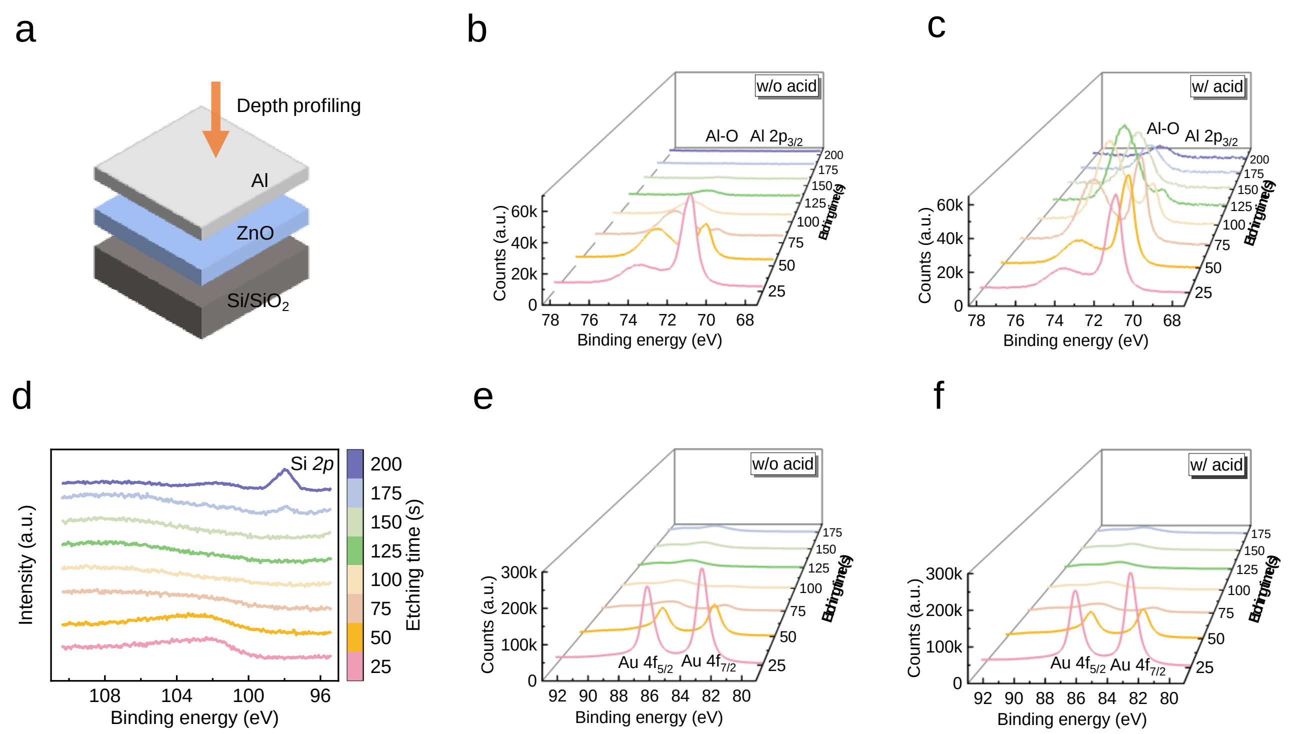

Figure S10. Metal diffusion under acid treatment. a, Sample structure of $Si/SiO_2/ZnO$ (~60 nm)/Al (~10 nm) and schematic of XPS depth profiling. Al 2p core level spectra at various depths for the samples **b**, without acid treatment and **c**, with acid treatment, **d**, Si 2p core level spectra for the acid-treated sample. Au 4f core level spectra at different depths for samples **e**, without and **f**, with acid treatment.

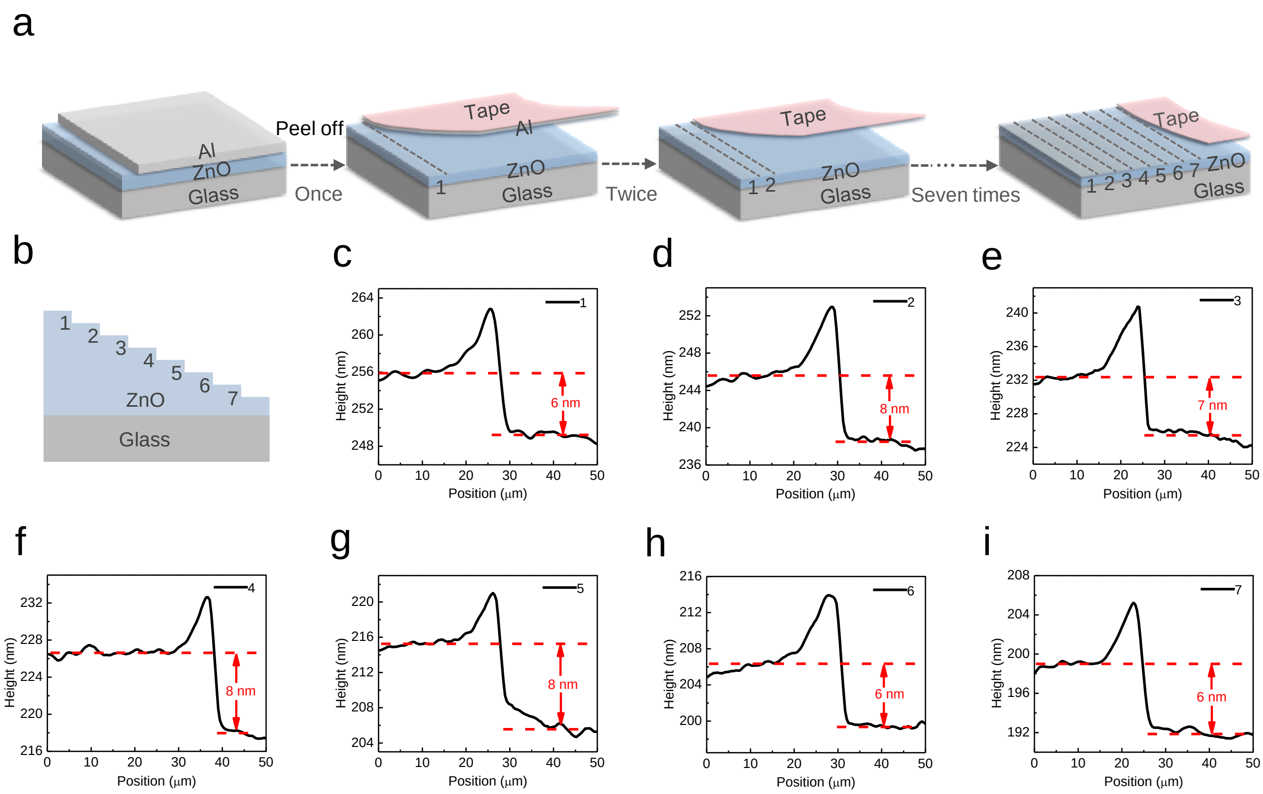

Figure S11. Sample preparation for the FTIR measurements. Schematic illustration for **a**, the multiple peeling process and **b**, cross-section of the sample. **c**-**i**, Thickness evaluation for the film after each peeling-off through AFM measurements.

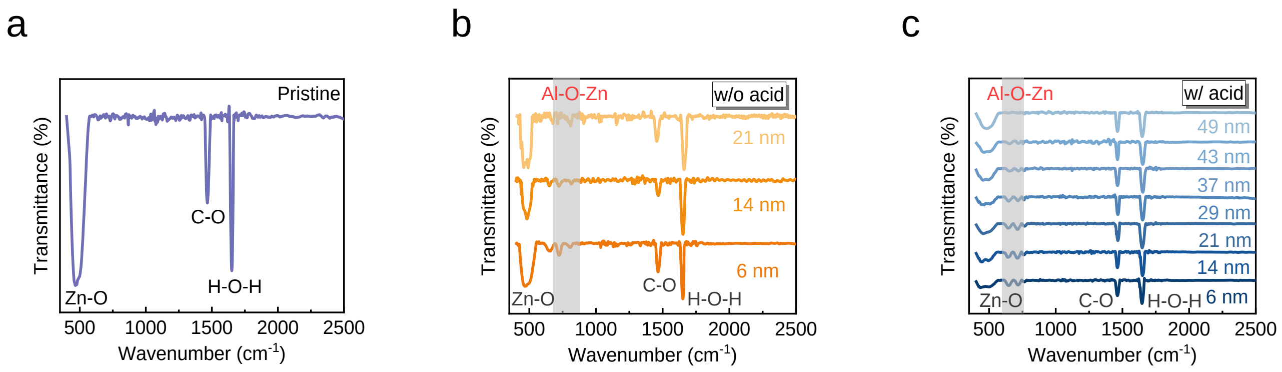

Figure S12. Fourier transformed infrared (FTIR) spectroscopy characterization. FTIR spectra of **a**, as-prepared ZnO film, Al-covered ZnO film **b**, without and **c** with acid treatment at different depths.

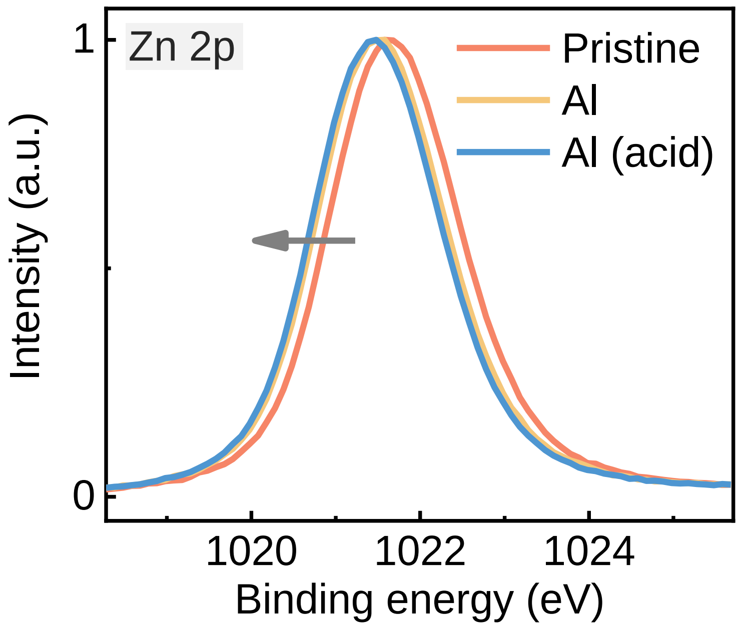

Figure S13. Zn 2p core level XPS spectra.

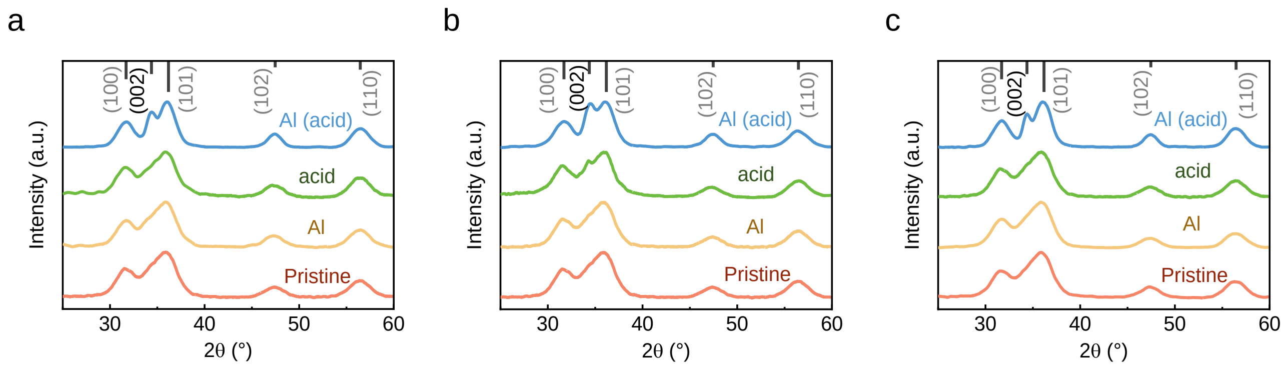

Figure S14. XRD patterns of four types of ZnO samples from three batches. The samples include pristine ZnO film, Al-deposited ZnO film (Al), ZnO film with acid treatment (acid), and Al-deposited ZnO film with acid treatment (Al (acid)).

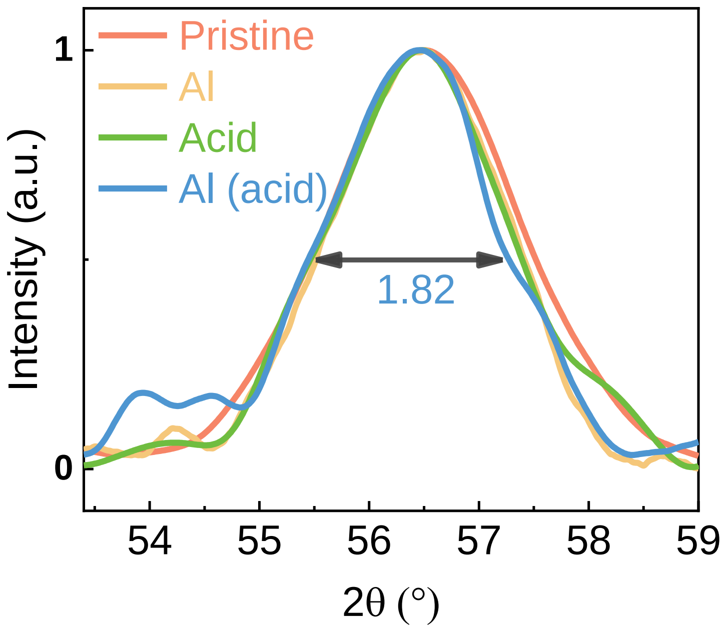

Figure S15. XRD patterns of ZnO films at (110) peak.

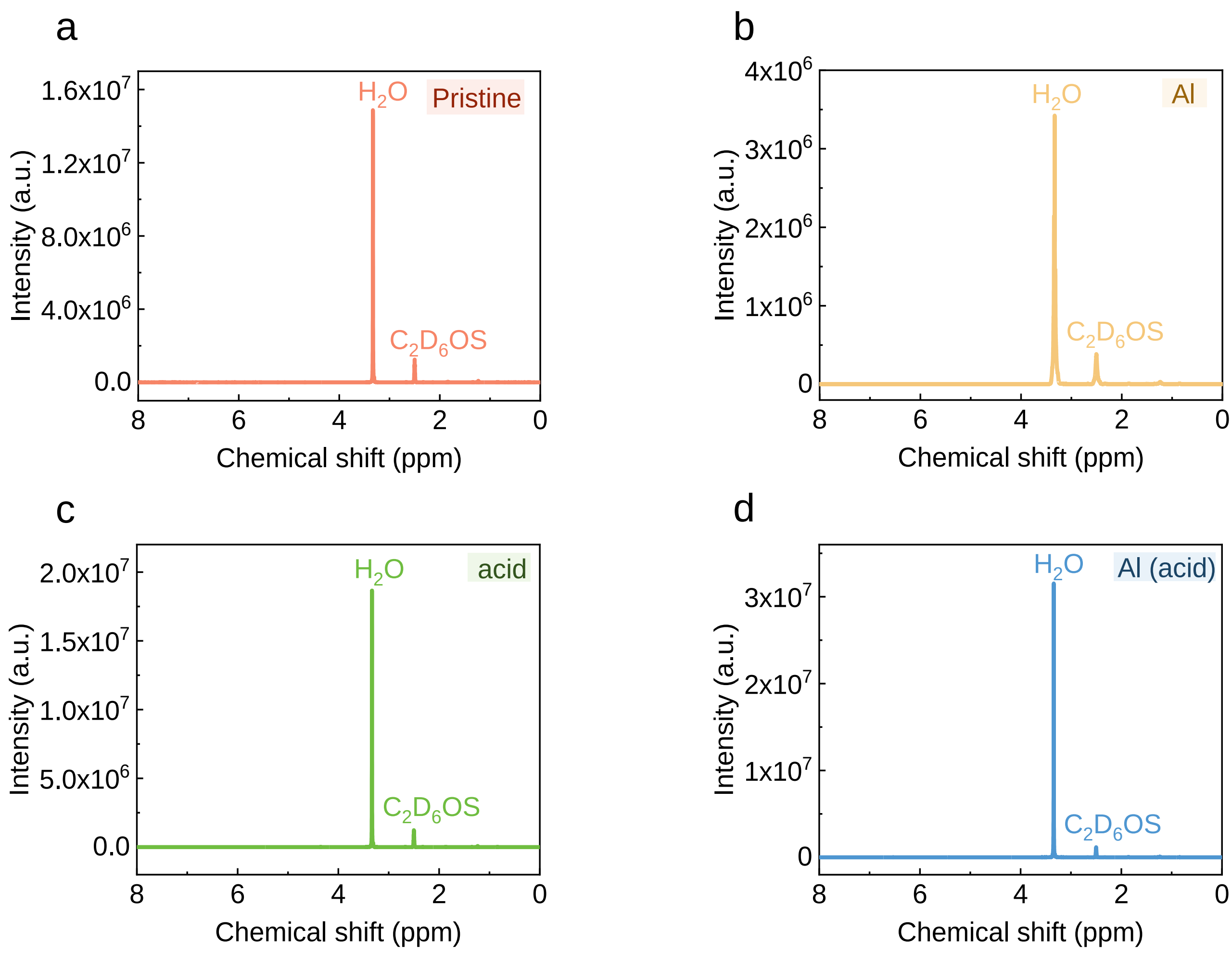

Figure S16. Nuclear magnetic resonance spectra of four samples. ^{1}H NMR spectra of ZnO thin films: **a**, as-prepared, **b**, Al-covered, **c**, acid-treated ZnO film and **d**, Al-covered ZnO film with acid treatment.

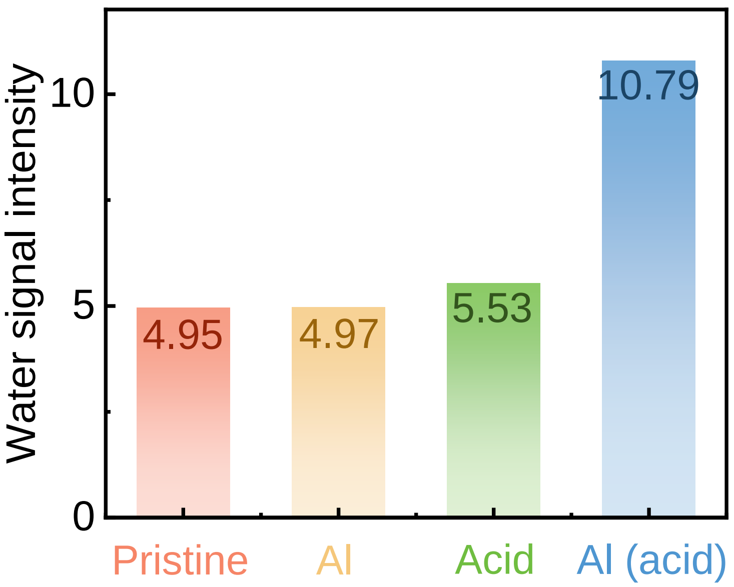

Figure S17. Intensity of water signal in the four samples.

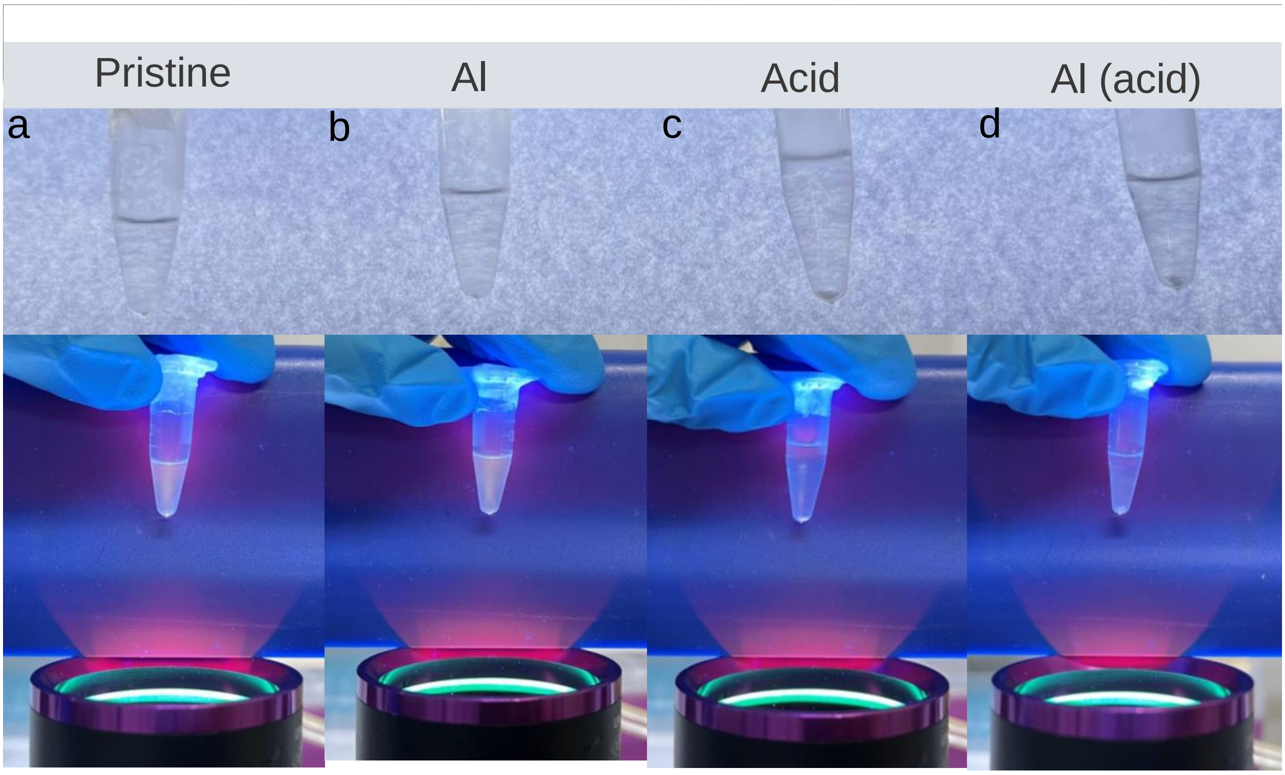

Figure S18. Redispersion of ZnO film in ethonal. Photographs of ZnO NPs exfoliated from **a**, pristine, **b**, ZnO/Al, **c**, acid-treated ZnO and **d**, acid-treated ZnO/Al films, dispersed in ethanol, ultrasonic treatment for two hours. The top panel photograph is under natural light and the bottom panel of the ZnO NPs are excited by a 365 nm UV flashlight.

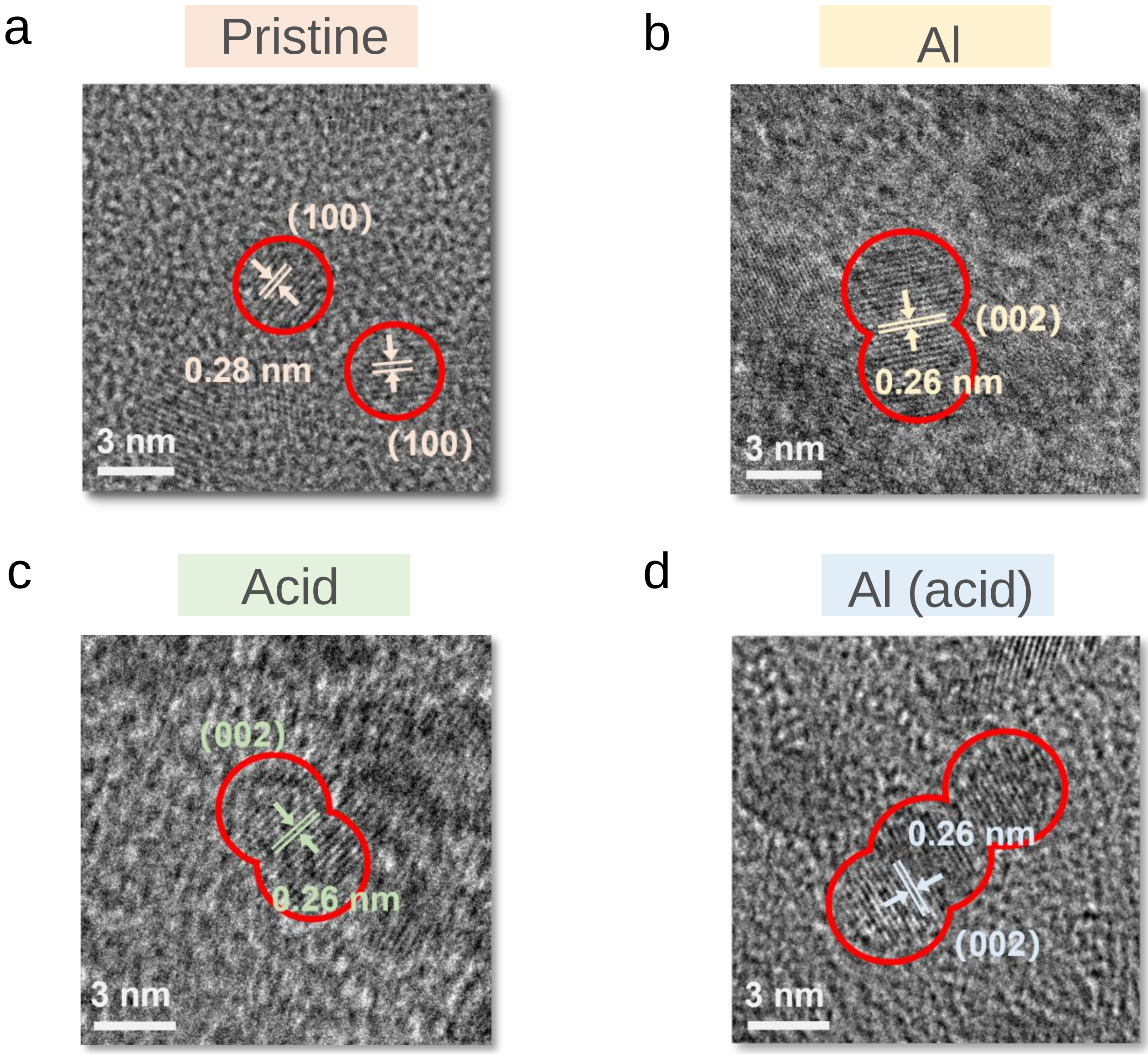

Figure S19. High-Resolution transmission electron microscopy images. HRTEM images of the four ZnO samples: **a**, as-prepared, **b**, Al-covered, **c**, acid-treated ZnO film, and **d**, Al-covered ZnO film with acid treatment.

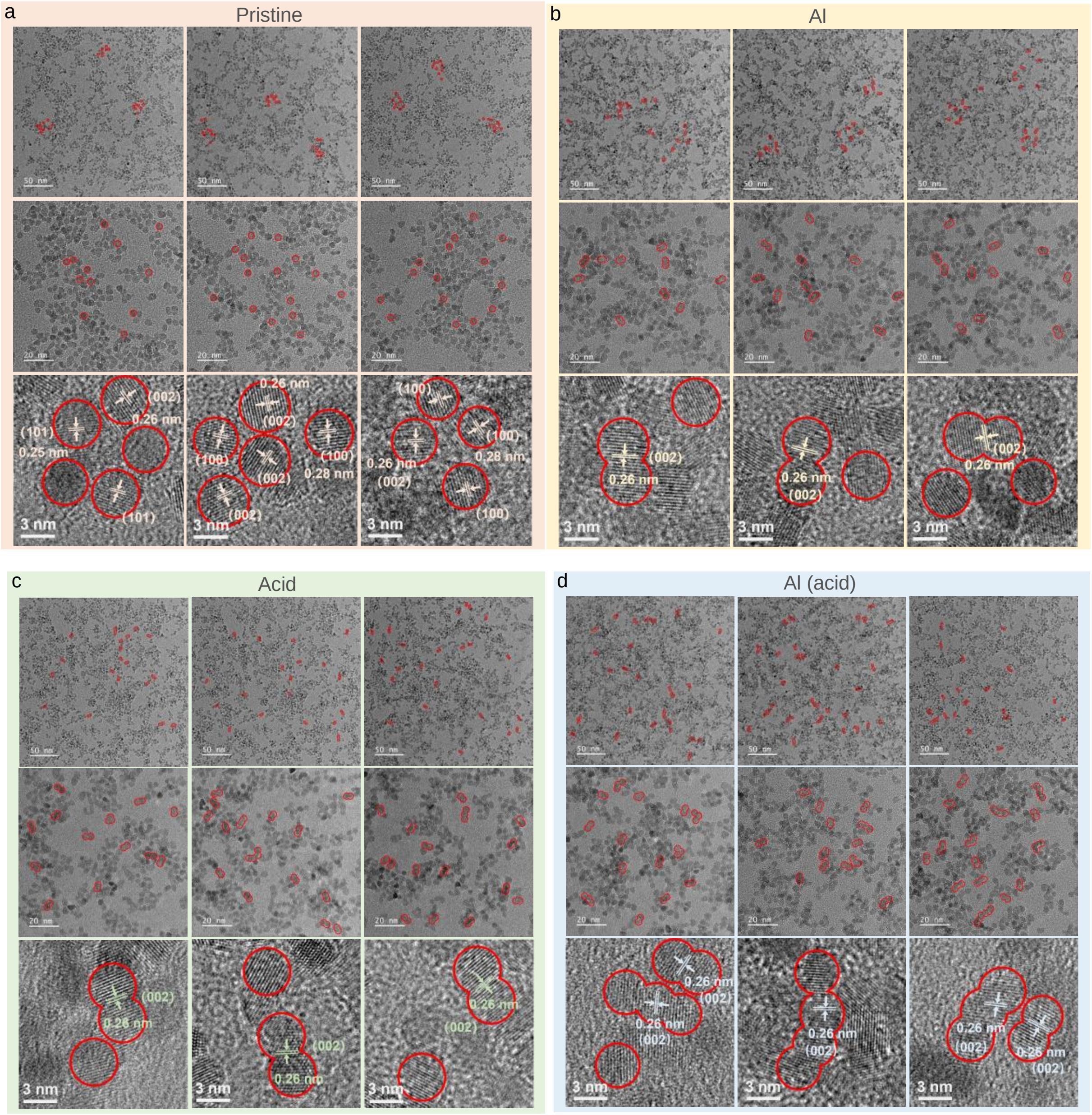

Figure S20. TEM images of four types of ZnO films from three batches. The samples include pristine ZnO, Al-deposited ZnO film (Al), ZnO film with acid treatment (Acid), and Al-deposited ZnO film with acid treatment (Al (acid)).

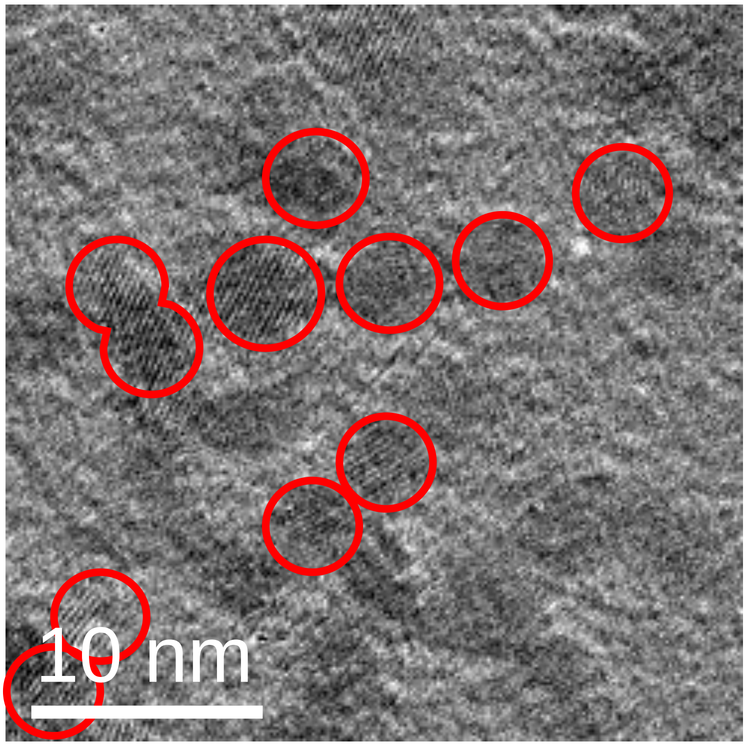

Figure S21. TEM image of Au-covered ZnO sample.

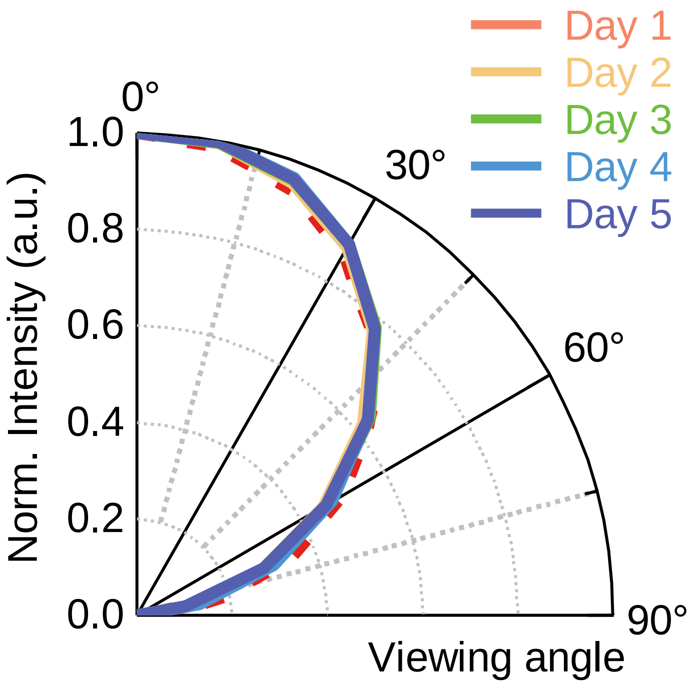

Figure S22. Angular distribution of light intensity over 5 days of positive ageing.

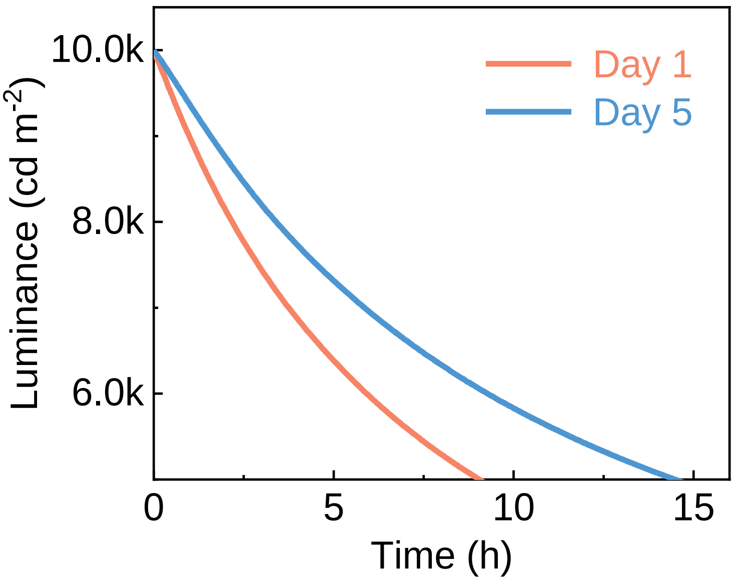

Figure S23. Operational stability of the QLEDs. Operational lifetime (T_{50}) of devices measured at an initial luminance of around 10,000 cd m^{-2} before and after ageing.

Table S1. Summaries of device performance for red CdSe-based devices.

Year	Device structure	Device performance		Ref.
		EL peak (nm)	EQE (%)	
2014	ITO/PEDOT:PSS/Poly-TPD/PVK/QDs/PMMA/ZnO/Ag	640	20.5	1
2015	ITO/PEDOT:PSS/TFB/QDs/ZnO/Al	625	12.0	2
2019	ITO/PEDOT:PSS/TFB/QDs/ZnO/Al	602	21.6	3
2019	ITO/PEDOT:PSS/TFB/QDs/ZnO/Al	602	30.9	4
2022	ITO/LZO/QDs/TCTA/NPB/HAT-CN/Ag	622	16.4	5
2022	ITO/PEDOT:PSS/TFB/QDs/ZnO/Al	628	17.4	6
2022	ITO/PEDOT:PSS/Poly-TPD/PVK/QDs/ZnO/Al	632	28.6	7
2022	ITO/PEDOT:PSS/TFB:CBP/QDs/ZMO/Ag	628	30.0	8
2023	ITO/PEDOT:PSS/TFB/QDs/ZnSnO/Al	628	15.5	9
2023	ITO/ZnO/TV-T2T/QDs/PCBP/TFB/PMAH/Al	628	20.2	10
2023	ITO/PEDOT:PSS/TFB/QDs/ZnO/Al	628	23.6	11
2024	ITO/PEDOT:PSS/TFB/QDs/PEABr/ZnMgO/Al	628	27.6	12
2025	ITO/PEDOT:PSS/TFB/QDs/ZnO/Al	628	33.7	**This work**

Table S2. Summary of the calculated parameters for ZnO thin films in devices.

$\mu = \frac{d}{(t_d - t_{RC})E}$	t_d (μs)	t_{RC} (μs)	Q_{ave} (C)	E (V m^{-1})	μ (cm^2 V^{-1} s^{-1})
**ZnO (w/o acid)**	0.30	0.14	5.00×10^{-8}	1.59×10^{8}	3.54×10^{-5}
**ZnO (w/ acid)**	0.20	0.12	2.82×10^{-10}	9.38×10^{5}	1.20×10^{-2}

The mobility was calculated using the corrected equation $\mu = \frac{d}{(t_d - t_{RC})E}$, where d is the thickness of the ZnO film, t_d is the TrEL delay time, t_{RC} is the charging time, and E is the electric field strength. To measure these parameters accurately, a 50 Ω series resistor was incorporated to measure transient current (see Fig. S7b-c). By fitting the transient current, the *RC* charging time (t_{RC}) and charge quantity were obtained using $Q(t) = \int_{t_{RC}}^{t} I(t)dt$. The average charge was calculated as $Q_{ave} = \frac{\int_{t_{RC}}^{t_d} Q(t)dt}{t_d - t_{RC}}$, and the corresponding average electric field strength was derived from $E = \frac{Q_{ave}}{S\varepsilon_0\varepsilon_r}$. Detailed results are provided in Table S2.

Table S3. Double-exponential fitting parameters for PL decay of QDs.

Sample	A_1	τ_1 (ns)	A_2	τ_2 (ns)	τ_{avg} (ns)
Glass	0.40	8.92	0.60	27.40	24.10
ZnO	0.72	10.38	0.28	16.30	12.62
ZnO/Al	0.74	9.30	0.26	15.57	11.62
ZnO (acid)	0.46	9.19	0.54	22.78	19.30
ZnO/Al (acid)	0.33	5.59	0.67	18.94	17.25
ZnO/Al (acid-5 days)	0.49	9.96	0.51	27.83	23.26

The average PL lifetime τ_{avg} of QDs in various devices was calculated using the following equation:

$$\tau_{avg} = \frac{A_1\tau_1^2 + A_2\tau_2^2}{A_1\tau_1 + A_2\tau_2}$$

Supplementary references

1. Dai, X. L. et al. Solution-processed, high-performance light-emitting diodes based on quantum dots. *Nature* **515**, 96-99 (2014).

2. Yang, Y. X. et al. High-efficiency light-emitting devices based on quantum dots with tailored nanostructures. *Nature Photonics* **9**, 259-266 (2015).

3. Shen, H. B. et al. Visible quantum dot light-emitting diodes with simultaneous high brightness and efficiency. *Nature Photonics* **13**, 192-197 (2019).

4. Song, J. J. et al. Over 30% external quantum efficiency light-emitting diodes by engineering quantum dot-assisted energy level match for hole transport layer. *Advanced Functional Materials* **29**, 1808377 (2019).

5. Jing, J. P. et al. Highly efficient inverted quantum dot light-emitting diodes employing sol-gel derived Li-doped ZnO as electron transport layer. *Organic Electronics* **103**, 106466 (2022).

6. Li, Y. F. et al. Charge balance in red QLEDs for high efficiency and stability via ionic liquid doping. *Advanced Functional Materials* **32**, 2203641 (2022).

7. Chen, H. T. et al. Controlling electron transport towards efficient all-solution-processed quantum dot light emitting diodes. *Journal of Materials Chemistry C* **10**, 8373-8380 (2022).

8. Fang, Y. F. et al. Highly efficient red quantum dot light-emitting diodes by balancing charge injection and transport. *ACS Applied Materials & Interfaces* **14**, 21263-21269 (2022).

9. Wang, T. et al. Sn-doped ZnO for efficient and stable quantum dot light-emitting diodes via a microchannel synthesis strategy. *Nanoscale* **15**, 18523-18530 (2023).

10. Xie, L. M. et al. High-performance inkjet-printed inverted QD-LEDs based on cross-linkable electron regulation layers. *Chemical Engineering Journal* **477**, 146789 (2023).

11. Jia, S. Q. et al. Optimizing ZnO–quantum dot interface with thiol as ligand modification for high-performance quantum dot light-emitting diodes. *Small* **20**, 2307298 (2023).

12. Chen, Q. Y. et al. Phenethylammonium bromide interlayer for high-performance red quantum-dot light emitting diodes. *Nanoscale Horizons* **9**, 465-471 (2024).
